# An integrative naturopathic oncology approach in metastatic malignant melanoma: two case reports

**DOI:** 10.3389/fonc.2026.1867929

**Published:** 2026-07-10

**Authors:** Sara Izadi-Najafabadi, Sydney Moffatt, Mackenzie Neufeld, Gurdev Parmar

**Affiliations:** 1 Research Department, The Parmar Foundation, White Rock, BC, Canada; 2 Research Department, Integrated Health Clinic, Surrey, BC, Canada

**Keywords:** case report, hyperthermia, integrative oncology, intravenous vitamin C, malignant melanoma, mistletoe therapy, Complementary Therapies, naturopathic care

## Abstract

**Introduction:**

Malignant melanoma is the most aggressive form of skin cancer, with increasing incidence and a high risk of metastasis and mortality. While conventional treatments such as surgery, immunotherapy, and targeted therapy remain standard, interest in integrative naturopathic oncology approaches is growing.

**Case representation:**

Case 1 is a woman in her early 60s who was initially diagnosed with malignant melanoma of the lower back in the early 1990s at age 30. This was treated with surgical excision alone, after which she remained in remission for nearly 2 decades. Between 2011 and 2018, she experienced multiple recurrences in the left axilla, paraspinal lumbar tissue, and gastric fundus. She underwent surgery for these recurrences and received stereotactic body radiation therapy (SBRT) to the spinal disease. Following each recurrence, she declined systemic therapy and pursued adjuvant naturopathic treatment cycles including fever-range whole-body hyperthermia (FR-WBHT), modulated electro-hyperthermia (mEHT), intravenous pharmacologic ascorbic acid, mistletoe therapy, repurposed drugs, and oral supplementation. Since her last recurrence in 2018, she has remained in clinical remission, with follow-up PET imaging through 2026 showing no evidence of disease.

Case 2 is a 65-year-old woman with recurrent melanoma, with metastases to the brain, liver, and lymph nodes. When no conventional treatment options were offered, she pursued naturopathic therapies including hyperthermia (mEHT, FR-WBHT), intravenous pharmacologic ascorbic acid, mistletoe therapy, repurposed drugs, and targeted supplementation. Imaging revealed gradual reductions in tumor size during treatment, marked by progressive decrease in masses. This resulted in complete remission by 2017, which remains in 2026.

**Conclusion:**

These cases describe prolonged remission temporally associated with multimodal integrative naturopathic oncology care in two cases of metastatic melanoma who also underwent surgery and/or radiotherapy. Alongside surgery, patients received hyperthermia, mistletoe extract, intravenous vitamin C, targeted pharmaceutical agents, and individualized supplementation. Both patients demonstrated regression of metastatic lesions with no detectable disease on imaging. Larger clinical studies are needed to further evaluate the safety and potential therapeutic effects of this approach in melanoma patients.

## Introduction

Malignant melanoma is the seventh most common cancer in the USA (1) and Canada (2), and has the highest mortality rate among invasive skin cancers (3). Overall 5-year survival for melanoma is around 94% (3) and 89% (2) in the USA and Canada respectively, reflecting excellent outcomes when detected in its earliest stages. When diagnosed at a distant metastatic stage, overall survival drops sharply to roughly 35% (3). The primary risk factors for malignant melanoma include excessive exposure to ultraviolet (UV) radiation, such as intense sun exposure or use of tanning beds, fair skin, light hair and eye color, genetic susceptibility, and immunosuppression (4–6).

Prognosis for malignant melanoma depends on factors such as the thickness of the primary tumor (Breslow depth), presence of ulcerations, the mitotic rate and the presence of metastatic spread (4). Treatment is completely stage-dependent and typically includes surgical resection, radiotherapy, immune checkpoint inhibitors, and targeted therapies for advanced disease (7). Since 2011, immune checkpoint inhibitors (ICIs) have revolutionized malignant melanoma treatment and prognosis. The approval of ipilimumab (a CTLA-4 inhibitor) (8) was followed by PD-1 inhibitors such as nivolumab and pembrolizumab (9–11), and more recently LAG-3 inhibitors (e.g. relatlimab) (12). For patients with BRAF V600 mutations, BRAF or MEK inhibitors (e.g., dabrafenib) have improved outcomes, with some patients achieving long-term survival (13, 14).

Despite improved survival outcomes, malignant melanoma survivors—particularly those treated with immune checkpoint inhibitors—often experience lower quality of life, toxicity burden, long-term symptoms such as fatigue, psychological distress, and unmet survivorship needs (15, 16). As such, many patients with advanced cancer—including those diagnosed with metastatic malignant melanoma—tend to use integrative oncology alongside standard care. Integrative oncology is defined as the patient-centered and evidence-based incorporation of nutrition, mind–body practices, nutraceuticals, lifestyle therapies and other modalities into cancer care (17–19).

Supportive care interventions have demonstrated potential benefits for patients with metastatic malignant melanoma. Multidisciplinary programs incorporating exercise, nutrition, psychological support, and complementary therapies have demonstrated feasibility, acceptability, and high patient engagement during treatment. In this report, we present two cases of metastatic malignant melanoma treated at the Integrated Health Clinic (IHC) with integrative naturopathic protocols, and no conventional systemic treatment. These case reports are documented in accordance with the CARE reporting guidelines (20) and present two cases of long-term remission in metastatic malignant melanoma following surgery (with or without radiation) and an integrative naturopathic oncology care protocol in the absence of systemic therapy.

## Patients’ information

This case report includes two women with malignant melanoma who later developed metastatic disease following initial treatment for localized primary tumors (**Table 1**). Case 1 is a woman diagnosed at age 30 in the early 1990s with a 1.85 mm Breslow depth melanoma of the lower back, treated with wide local excision and clear margins. Sentinel lymph node biopsy was not performed, and no adjuvant therapy was recommended at diagnosis. She remained disease-free for approximately 18 years before developing BRAF-positive metastatic melanoma with multiple recurrences between 2011 and 2018, involving the left axilla, paraspinal tissue, and gastric fundus. **Supplementary Figure 1A** shows a timeline of malignant melanoma recurrences and the treatments Case 1 received between 2012 and 2018.

**Table 1:** Patients’ demographic and clinical characteristics.

Characteristic	Case 1	Case 2	
Sex	Woman	Woman	
Initial diagnosis year	Early 1990s	Early 2010s	
Age at diagnosis	30	50	
Primary tumor site	Lower back	Posterior right scalp	
Initial pathology	Breslow depth 1.75 mm	Breslow depth 5 mm	
Initial treatment	Local excision	Local excision	
Adjuvant therapy	No	No	
Disease-free interval after initial treatment	~18 years	~2 years	
Sites of metastasis	• 2012: Left axilla • 2014: Paraspinal lumbar tissue • 2018: Gastric fundus	• 2013: Brain • 2014: Liver & lymph nodes	
Lifestyle history	No tobacco use Occasional alcohol Occasional marijuana	No tobacco use No alcohol use	
Past medical history	Not significant	Fatty liver, Hashimoto’s thyroiditis (on Synthroid 150 mcg), benign thyroid nodule, Transient vesicular-like rash in early 2020s	
Family history	Colon cancer (paternal grandmother); breast cancer (maternal aunt)	Lymphoma and arthritis (father)	
Clinical course and diagnostic findings			
First Recurrence	2012: Left axilla (Stage III) • Excisional biopsy: BRAF V600 stage III melanoma (left axilla), PDL1+ • PET/CT: 5 regional metastases (SUV max 1.4-7.1, 2.4 cm in maximum dimension). • Post-surgical pathology: 7/12 nodes positive; o ECE; S-100+. Melan-A+, HMB-4+. o Clear margins. • LDH: 131-148	2013: Brain • Excisional biopsy: invasive melanoma (Breslow depth of 10 mm in anterior right temporal scalp, 9mm), o Deep margin clearance of 1 mm • CT scan: lesions in right parietal region (1.4 cm) and left basal frontal area (5 mm)	
Second Recurrence	2014: Paraspinal lumbar tissue (Stage IV) • PET/CT: left paraspinal at L4-L5 (SUV 4.1, 2.7 cm). • Biopsy: stage IV melanoma o S-100+, Melan-A+, CKAE1/AE3- and 00138-	2014: Liver & lymph nodes (Stage IV) • PET/CT: preauricular/cervical nodes (SUV 9.6, 1.2 cm); suspicious porta hepatis and brain lesions (SUV 14.8, 3.3 cm). • Biopsy: BRAF+ invasive metastatic melanoma (right preauricular lymph node). o Other mutations negative • MRI: No evidence of cerebral metastases • ECOG= 0 • LDH: 313	
Third Recurrence	2018: Gastric fundus (Stage IV) • PET/CT: gastric fundus lesion (SUV 7.2, 4.5 cm). • Biopsy: S-100+ melanoma o Clear margins. • ECOG=1	N/A	

BRAF, B-Raf proto-oncogene, serine/threonine kinase; CT, Computed Tomography; ECE, Extracapsular Extension; FDG, fluorodeoxyglucose; LDH, lactate dehydrogenase; ECOG, Eastern Cooperative Oncology Group; PET, Positron Emission Tomography; MRI, Magnetic Resonance Imaging; PLD1, programmed-death ligand 1; SUV, Standard Uptake Volume.

Case 2 is a woman diagnosed in her mid-50s in the early 2010s with a 5 mm Breslow depth melanoma of the posterior right scalp. She underwent wide local excision with negative sentinel lymph nodes and did not receive adjuvant therapy. Despite initial no evidence of disease on surveillance imaging, she developed metastatic melanoma involving the brain, liver, and lymph nodes 2 years following her initial diagnosis.

Both patients had no history of tobacco use, and neither received adjuvant systemic therapy at initial diagnosis. **Supplementary Figure 1B** shows a timeline of malignant melanoma recurrence for Case 2.

## Diagnostic assessment (Recurrences)

**Figure 1:**
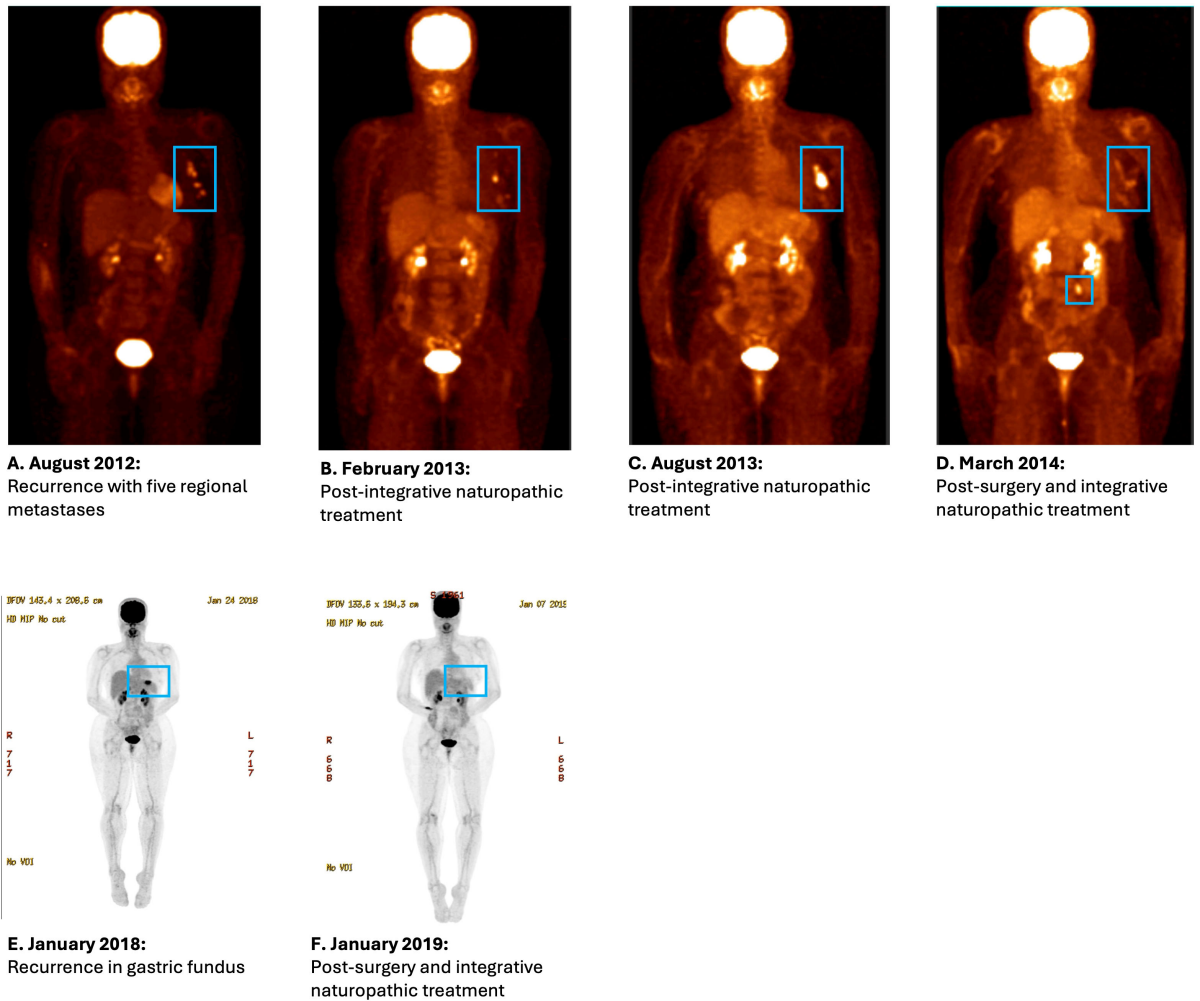
**(A–F).** Case 1’s PET scans showing disease progression and response to integrative treatments over time (2012-2019).

Case 1 experienced three confirmed recurrences of BRAF-positive malignant melanoma following her initial diagnosis in 1990s. In 2012, the patient experienced her first recurrence following excisional biopsy of a left axillary mass, which confirmed BRAF-positive, programmed-death ligand 1-positive (PDL1+), HMB-45+, S-100+, Melan A, stage III metastatic malignant melanoma with five regional metastatic deposits, and a maximum standardized uptake value (SUV max) of 7.1 in one nodule (**Figure 1A**). After this recurrence, the patient was given an estimated life expectancy of less than one year and was referred for disability support services. **Table 1** shows a chronological summary of the diagnostic assessments and corresponding results for both cases.

Despite the poor prognosis, Case 1 survived an additional two years and experienced a second and third recurrences in 2014 and 2018. Following the onset of new lower back pain, imaging confirmed a metastatic S-100+, Melan-A+ lesion at L4–L5 (SUVmax 4.1; **Figure 1D**) in 2014. The third recurrence was identified in 2018 during routine surveillance, when a PET scan detected a hypermetabolic S-100+ lesion in the gastric fundus (**Figure 1E**), which biopsy confirmed as recurrent metastatic malignant melanoma.

Case 2 experienced two confirmed recurrences of metastatic melanoma following initial diagnosis. Her first recurrence was identified in 2013 with a new primary scalp lesion (anterior right temporal scalp) confirmed as invasive melanoma, alongside imaging evidence of metastases involving the brain, liver, and lymph nodes. A second recurrence was confirmed in early 2014 through PET/CT and biopsy of an FDG-avid right preauricular lymph node.

## Therapeutic intervention

Across their disease courses, both Case 1 and Case 2 pursued integrative oncology approaches, alongside selected conventional intervention, at the Integrative Health Clinic (IHC), which included the following modalities (**Table 2**):

**Table 2:** Patients’ therapeutic intervention.

Case	Year	Clinical event	Conventional management	Integrative treatments
Case 1	2012	Axillary recurrence	- Excisional biopsy - Declined systemic therapy (i.e., Ipilimumab and Interleukin) - Palliative care recommended	51 mEHT sessions; 16 FR-WBHT sessions; 51 High-dose ascorbic acid Mistletoe therapy Targeted supplementation
	2014	Paraspinal/lumbar recurrence	- Subtotal resection - SBRT 45 Gy in 3 fractions - Declined systemic therapy (i.e., interferon and interleukin 2)	1 mEHT session; Targeted supplementation
	2018	Gastric fundus recurrence	- Surgical resection	11 mEHT sessions 3 FR-WBHT sessions 6 High-dose ascorbic acid Mistletoe therapy Targeted supplementation
	2023	Supportive care (no evidence of disease)	NA	1 Preventive FR-WBHT session; Lifestyle management; Mistletoe therapy Targeted supplementation
Case 2	2013	Scalp recurrence	- Surgical resection - no other treatment was offered due to disease regression	16 mEHT; 3 FR-WBHT; 71 High-dose ascorbic acid Mistletoe therapy Targeted supplementation

FR-WBHT, fever-range whole body hyperthermia; mEHT, modulated electrohyperthermia; NA, not applicable.

- modulated electro-hyperthermia (mEHT)
- fever-range whole-body hyperthermia (FR-WBHT)
- high-dose ascorbic acid
- mistletoe therapy
- lifestyle modifications
- targeted oral supplementation

Case 1 experienced three recurrences, each managed initially with surgery followed by integrative oncology, while consistently declining further conventional systemic therapy including targeted therapy and immunotherapy (**Table 2**). The first recurrence (2012) involved a left axillary lesion treated with excisional biopsy and subsequent resection of residual tumor and lymph nodes; adjuvant systemic therapies, including Ipilimumab and Interleukin, were declined, and she instead underwent naturopathic care including 51 mEHT and 16 FR-WBHT sessions. The second recurrence (2014) presented as spinal metastasis at L4–L5, managed with subtotal surgical resection due to anatomical constraints, followed by SBRT (45 Gy in 3 fractions); she declined immunotherapy including interferon and interleukin 2 and received one additional mEHT session over the radiated lumbar field in October 2016 and supportive supplements including tamoxifen, niacin, and BCQ (Boswellia, Curcumin, Quercetin) before each SBRT radiation session.

The third recurrence (2018) involved gastric fundus metastasis, treated with surgical resection without complications, followed by additional integrative treatments including 3 FR-WBHT sessions, 11 mEHT session, 6 IVAA sessions, and 5 IV Helixor mistletoe sessions. Across all recurrences, the patient consistently refused further conventional systemic therapy in favor of integrative approaches. All supplements and IV therapies continued until September 2018. By then, she had received treatments which were continued beyond confirmed remission in 2015.

Case 2 underwent surgical excision of a right temporal scalp lesion with deep margin clearance of 1 mm in 2013. Following evidence of second recurrent and widespread metastatic melanoma in January 2014, IHC recommended a holistic naturopathic palliative treatment program consisting of a low glycemic index whole foods diet, walking at least four times a week, daily mindfulness meditation, as well as a repurposed drug and nutraceutical protocol for her. Beginning in February 2014, she underwent a three-month integrative naturopathic oncology care protocol that included: 3 FR-WBHT sessions, 16 mEHT sessions, 71 IVAA sessions, and IV Helixor mistletoe sessions (**Table 2**).

Given the overall pattern of disease regression between January and May 2014, treating conventional care providers noted significant uncertainty regarding the optimal management strategy, including whether to observe the patient, initiate systemic therapy for metastatic disease, or pursue further surgical intervention with neck dissection and re-excision of residual disease. Due to the absence of clear evidence to guide treatment in this setting and Case 2’s clinical stability, a decision was made to continue close observation with interval imaging. It was noted that should clinically meaningful disease progression occur, systemic treatment options, including chemotherapy (i.e., Dacarbazine), immunotherapy (e.g., Ipilimumab), and BRAF inhibitors remained available.

Both patients received a similar targeted supplementation regimen, including CoQ10, Celebrex, fermented wheat germ extract, desiccated thyroid, intravenous ascorbic acid, Vitamin D, melatonin, and alpha lipoic acid with/without the addition of dichloroacetate (DCA). For more information, please see **Supplementary Table 1**.

Throughout treatment, both cases generally tolerated IHC treatment protocols, except Case 2 that developed mild headache following IVAA. She also developed arthritis in her hands and feet as well as myalgias and episcleritis. In July 2014, rheumatology evaluation suggested inflammatory arthritis or a paraneoplastic phenomenon. Interestingly and notably, these symptoms improved after discontinuation of the Can-Arrest formulation, which was used for a short duration of time but quickly ceased given the patient’s arthritic reaction.

## Follow-up and outcomes

Case 1 and 2 experienced favorable clinical outcomes, with an extended survival, tumor regression, and decreased metabolic response far beyond initial expectations.

### Survival

Both cases were alive at the time of writing. Case 1 has survived approximately 14 years since her first metastatic recurrence in 2012, and Case 2 approximately 13 years since her first recurrence in 2013, indicating prolonged survival despite metastatic disease, absence of systemic therapy, and an initially poor prognosis. Case 1 achieved a disease-free progression interval of approximately 7 years (2019–2026). Case 2 achieved and maintained a durable complete remission, with a disease-free progression interval of approximately 9 years (2017–2026) (**Supplementary Figure 2**).

### Tumor regression

In Feb 2012, Case 1 demonstrated a 35% reduction in tumor burden after three months of integrative naturopathic oncology care, with several lesions decreasing in size (**Figure 1B****).** Over one year after initiating naturopathic treatment, the largest nodules decreased from 4.8 cm to 0.7 cm. There was no reported evidence of tumor reduction following the second and third recurrences for Case 1.

**Figure 2:**
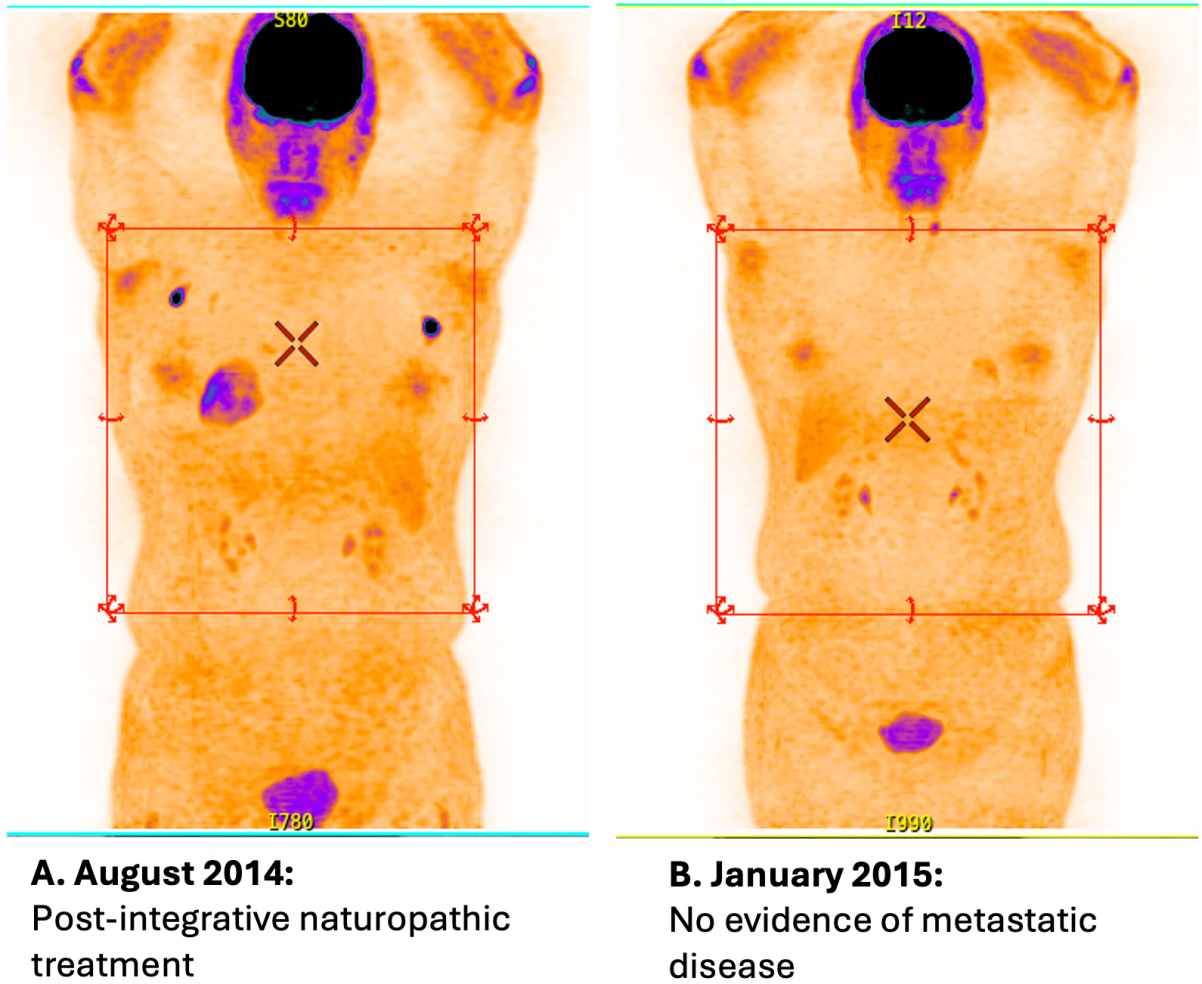
**(A, B)**. PET scans showing Case 2’s response to integrative treatments over time (2014-2015).

In Case 2, PET/CT imaging in August 2014 showed marked reduction in cervical lymph nodes from 1.2 cm to approximately 0.5 cm (**Figure 2A**) followed by complete radiographic resolution of metastatic disease by January 2015 (**Figure 2B**). This outcome was described by the treating oncologist as a “spontaneous” or “miraculous” remission. However, it is important to note that the patient had been undergoing an intensive integrative naturopathic oncology regimen from February 2014 through September 2018.

### Metabolic response

For Case 1, serial PET/CT imaging demonstrated an initial partial metabolic response following treatment as evidenced by reduced FDG uptake on the February 2013 scan compared with baseline imaging in August 2012 (**Figures 1A, B**). FDG uptake decreased across previously identified metastatic sites, including the dominant left axillary lesion (SUVmax 7.1→4.9), lower chest wall lesion (SUVmax 2.4→1.6), and upper left axillary lymph node (SUVmax 1.6→1.4).

In August 2013, a restaging PET/CT scan revealed complete resolution of one tumor (**Figure 1C**); however, two adjoining hypermetabolic nodules in the left chest wall adjacent to an intercostal space demonstrated increased metabolic activity, with an SUV Max rising from 5.1 to 7.7, suggesting persistent metabolic activity in that region.

By March 2014, imaging demonstrated a complete metabolic response was observed in the left axilla, with no residual FDG uptake, indicating no evidence of active disease at that site (**Figure 1D**). Following these results, she did not proceed with the recommendation for a significant lymphadenectomy of the left axilla or the prescribed systemic chemotherapy.

Subsequent recurrences in the spine (2014; SUV 4.1→2.9 post-treatment) and gastric fundus (2018; SUVmax 7.2→no abnormal uptake post-treatment) (**Figures 1E, F**) each achieved complete response following combined surgical and integrative treatment. From 2020 to 2026, serial imaging confirmed a sustained absence of disease.

For Case 2, serial PET/CT imaging demonstrated an initial favorable metabolic response, with near resolution of FDG-avid disease in the liver, porta hepatis, and cervical lymph nodes by August 2014 (**Figure 2A**) alongside a marked reduction in previously hypermetabolic lesions (e.g., SUVmax reduction from 9.6 to 3). However, new bilateral FDG-avid axillary lymphadenopathy was observed. The largest lymph node on the right measured 1.8 × 2.6 cm with a SUV max of 19.6, while the largest on the left measured 1.8 × 2. 4 cm with a SUV max of 16.2. Follow-up PET imaging demonstrated transient, fluctuating FDG-avid findings, including non-specific subcutaneous lesions in 2015 and cervical lymphadenopathy in 2016, which subsequently resolved. By May 2017, PET imaging showed no evidence of metastatic melanoma, with annual surveillance scans through 2026 confirming sustained remission and no recurrence.

## Discussion

These two cases of recurrent stage IV malignant melanoma illustrate the long-lasting clinical responses in patients who decline systemic chemotherapy following surgery and naturopathic oncology protocols. Despite poor prognostic features such as metastatic disease, multiple recurrences, and expected limited survival, both patients achieved complete remissions. An integrative oncology approach was used alongside conventional care (surgery and radiation), including adjunctive modalities such as hyperthermia, intravenous therapies, mistletoe, and targeted supplementation, with minimal reported side effects. These case reports generate a hypothesis for future research into the potential effectiveness and safety of integrative oncology approaches in a broader clinical context.

Both cases demonstrate shared therapeutic themes:

1. Multimodal Synergy: Each patient underwent coordinated integrative regimens that included FR-WBHT, mEHT, IVAA, mistletoe therapy, and targeted supplementation.
2. Treatment Individualization: The therapeutic approach was tailored not only to tumor burden and location but also to comorbidities, and treatment preferences. For example, Case 2’s protocol was changed when she developed inflammatory arthritis likely triggered by Can-Arrest supplements, the offending agent was promptly withdrawn with symptomatic resolution.
3. Sustained Remission Without Systemic Therapy: Neither patient received immune checkpoint inhibitors, BRAF/MEK inhibitors, or chemotherapy—standard treatments known to prolong survival in metastatic malignant melanoma.
4. Low Toxicity and High Tolerability: Across both cases, only Case 2 reported transient side effects/symptoms such as headache after IVAA.

The integrative treatment approaches used in Case 1 and Case 2 were designed to target multiple hallmarks of tumor development and progression. Cancer evades immune detection (21), induces angiogenesis (22), promotes inflammation (23), and alters cellular signaling to support growth and metastasis (24). In light of these complex and interconnected mechanisms, a multimodal integrative treatment strategy was pursued to target multiple biological pathways simultaneously.

Each component of the protocol was chosen with clear clinical rationale. mEHT was incorporated because of evidence suggesting it may sensitize cancer cells to standard therapies, including radiotherapy, and exert direct cytotoxic effects (25–32). VAA and mistletoe therapy were included based on reports of potential anticancer and immunomodulatory effect (33, 34). FR-WBHT was selected for its proposed immunomodulatory effects and potential to influence antitumor immune responses (35). Repurposed medications, oral supplements, and dietary interventions were incorporated with the aim of supporting overall patient health. Because many elements of the integrative oncology protocol for the two cases were intended to support immune function, they could theoretically have influenced immune-mediated tumor control, but this remains speculative and cannot be distinguished from spontaneous or treatment-related regression on the basis of these cases alone.

At the same time, it is important to note that the literature supporting IV vitamin C, mistletoe, and hyperthermia is not uniform: evidence for IV vitamin C remains preliminary (36), mistletoe studies are limited by design weaknesses and inconsistent survival findings (37), and hyperthermia continues to be debated because trial quality and clinical benefit remain uncertain (38). This uncertainty is particularly relevant in melanoma, where spontaneous regression, immune-mediated tumor control, and durable responses after surgery and/or radiotherapy alone are all recognized phenomena (39–41).

For this reason, the temporal association between integrative treatment and outcome should be interpreted cautiously, as the observed remissions may reflect factors other than the naturopathic protocol itself. For instance, immune activation following fever, vaccination, surgical resection or localized radiotherapy may contribute to systemic tumor control, including through mechanisms such as antigen release, and, in rare cases, abscopal effects (39, 42). Patients with oligometastatic melanoma may also experience prolonged disease control following surgery and/or radiotherapy alone (43, 44).

Therefore, these observations remain hypothesis-generating and do not permit conclusions regarding efficacy, as alternative explanations—including spontaneous or immune-mediated regression and effects of prior conventional treatments—cannot be excluded.

Hyperthermia shows promise in treating malignant melanoma. Clinical studies involving patients with malignant melanoma have also reported associations between combining hyperthermia with radiotherapy results and enhanced tumor response compared with radiotherapy alone (32, 45), which are all aligned with the results we have seen in our two cases. More specifically, mEHT is proposed to selectively impact tumor tissues by inducing cellular stress and apoptosis, while minimizing harm to surrounding healthy cells (46).

Fever is a well-known biological response of the innate immune system to infection or inflammation. When a fever is artificially induced, such as through FR-WBHT, a similar stimulation of the immune system is observed (47, 48) Clinical observations suggest that this immune activation may translate into therapeutic benefit. A case report of a patient with advanced malignant melanoma revealed that spontaneous episodes of fever—left untreated—were followed by tumor regression or stabilization, despite minimal conventional treatment. This unexpected disease course suggests that fever may stimulate immune responses capable of altering malignant melanoma progression, supporting emerging perspectives on the potential therapeutic value of fever in oncology (49). These findings are consistent with our hypothesis regarding the use of fever-range hyperthermia as a therapeutic modality to improve outcomes in patients with malignant melanoma.

Further support comes from immunotherapy studies showing that treatment-induced fever may enhance survival outcomes of metastatic malignant melanoma patients, and that suppressing fever could diminish the effectiveness of immune-based therapies (35, 50, 51). These insights may help explain why artificially induced fever could also benefit patients with malignant melanoma.

Preclinical and early translational studies suggest a potential role for high-dose ascorbate in melanoma. *In vitro* data (single study) report dose-dependent cytotoxicity in malignant melanoma cells via a pro-oxidant mechanism, while animal models demonstrate enhanced antitumor effects in combination with BRAF inhibition (33, 52). Clinical observations also indicate lower plasma vitamin C levels in patients with metastatic melanoma, which may be relevant to tumor–ascorbate interactions (53).

Mistletoe, or viscum album, is prepared using white-berried mistletoe from different host trees, each with its unique chemical profile and effects. Mistletoe have been reported to modulate immune activity by affecting multiple immune cells and cytokines, such as B and T cells, natural killer cells (34, 54). Clinical studies have described improvements in patient-reported outcomes, including fatigue, appetite, nausea, and pain (55). In the context of malignant melanoma, isolated case reports have described favorable clinical outcomes following mistletoe-based therapies. One report documented complete remission and long-term disease stability in a patient with metastatic melanoma treated with low-dose mistletoe extract as the sole adjuvant intervention (56), while another reported complete remission and prolonged survival following high-dose, fever-inducing Viscum album extract (57). Collectively, these reports are limited in scope but suggest similar individual cases of prolonged disease control that have been described in the literature.

Taken together, these two cases illustrate the possibility of prolonged disease control and complete remission in recurrent stage IV malignant melanoma following surgery and individualized integrative naturopathic oncology care, despite the absence of systemic chemotherapy or immunotherapy. These cases should be interpreted as descriptive and hypothesis-generating, warranting further prospective study of integrative oncology approaches in advanced melanoma.

Further research, including larger prospective clinical studies and controlled trials, is needed to evaluate the safety, tolerability, and potential clinical benefits of this approach in melanoma patients. Future investigations should assess objective clinical outcomes, including tumor response, disease progression, survival outcomes, quality of life measures, and potential interactions with standard-of-care therapies.

Moreover, artificial intelligence (AI) has emerging potential to enhance the field of oncology by supporting early risk assessment, metastasis detection, and personalized treatment planning. AI-based image analysis, including whole-slide imaging and image-based modeling, may help predict metastatic risk that may complement conventional assessment (58). Earlier identification of metastatic progression may help guide individualized care and optimize the integration of evidence-based natural therapies with conventional treatments. Future research may also explore AI as a tool to predict treatment response, monitor patient progress, and evaluate whether integrative oncology approaches are associated with improved outcomes.

## Limitations

The retrospective and descriptive nature of these reports, together with the substantial heterogeneity of the multimodal interventions, precludes causal inference. The intervention combinations were not standardized or controlled, limiting reproducibility and generalizability. The complexity of the treatment approach limits the ability to identify individual treatment components contributing to outcomes, and the contribution of individual modalities cannot be isolated. In addition, potential confounding factors, including surgical debulking, spontaneous regression, natural disease variability, and possible lesion misclassification, cannot be excluded. While spontaneous regression cannot be ruled out, the observed temporal associations remain descriptive in nature. Accordingly, these findings should be interpreted as exploratory and hypothesis-generating only, underscoring the need for well-designed observational and interventional studies to evaluate safety, efficacy, and underlying mechanisms.

## Data Availability

The data analyzed in this study is subject to the following licenses/restrictions: The data supporting the findings of this case report are derived from patient medical records and are not publicly available to protect patient privacy. Further details may be available from the corresponding author on reasonable request. Requests to access these datasets should be directed to research@theparmarfoundation.ca.
