# Supplementary material for: An integrative naturopathic oncology approach in metastatic malignant melanoma: two case reports
Source: Front Oncol. 2026 Jul 10;16:1867929. doi: 10.3389/fonc.2026.1867929 (PMC13395597; doi:10.3389/fonc.2026.1867929)
Supplement: Supplementary file 1 [file DataSheet1.docx]

**Supplementary Table 1.** Common targeted supplementation (nutraceuticals and pharmaceuticals products) for both patients

| **Product** | **Case 1 Dose / Frequency / Route** | **Case 2 Dose / Frequency / Route** |
| --- | --- | --- |
| CoQ10 | 100 mg; twice daily; oral | 100 mg; twice daily; oral |
| Celebrex* | 100 mg; twice daily; oral | 100 mg; twice daily; oral |
| Vitamin D | 5,000–10,000 IU; once daily; oral | 4,000 IU; twice daily; oral |
| Melatonin | 10–20 mg; bedtime; oral | 10 mg; bedtime; oral |
| Fermented wheat germ extract | 1 sachet daily; oral | 1 sachet daily; oral |
| Thyroid (desiccated) | 30 mg daily; oral | 30 mg daily; oral |
| Ascorbic acid | 25–50 g; once/twice weekly; IV | 25 g; twice weekly; IV |
| Alpha lipoic acid | 300 mg; once/twice weekly; IV | with DCA; twice weekly; IV |
| Mistletoe | 50–150 mg; twice weekly; SC  2000 mg; twice weekly; IV | 0.1–10 mg; twice weekly; IV |

CoQ10, Coenzyme Q10; DCA, Dichloroacetate; EGCG,Epigallocatechin gallate; IU, international unit; IV, intravenous; SC, subcutenaous.

***** Repurposed pharmaceuticals.

1. Case 1’s recurrences and treatments (2012–2018)


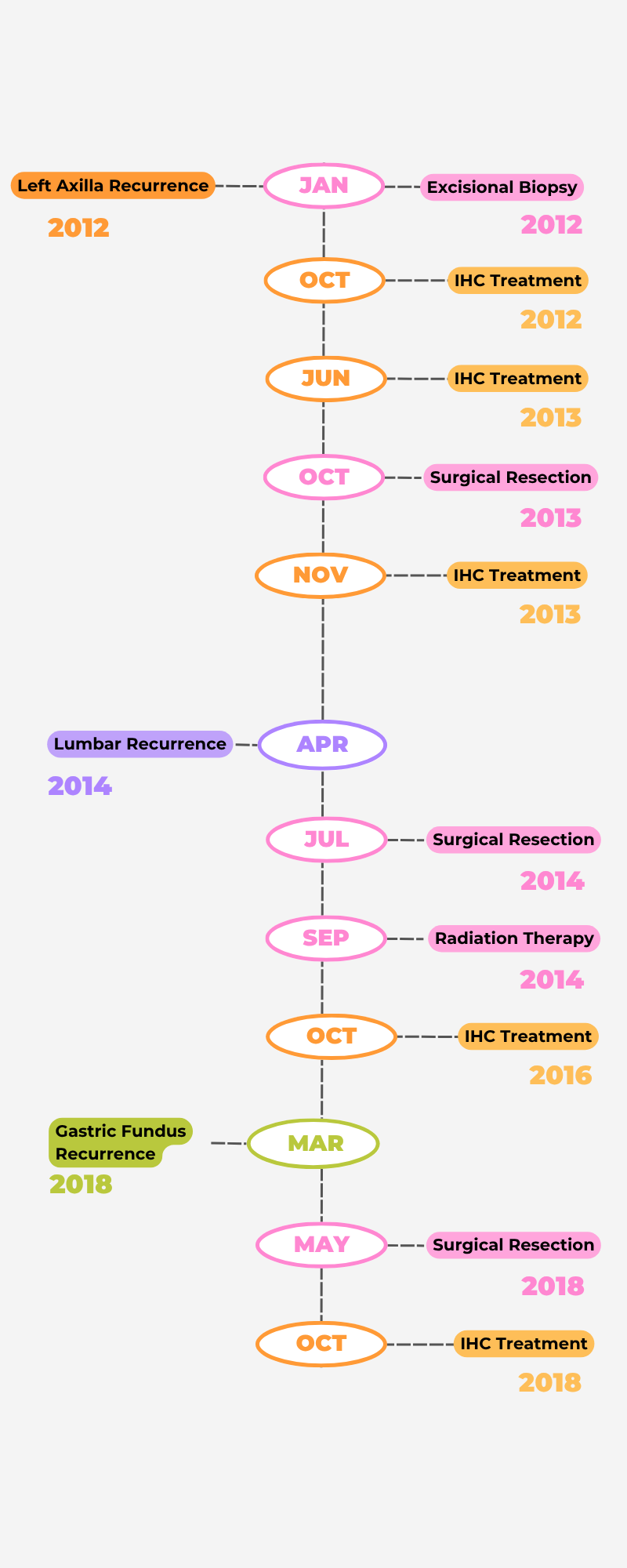

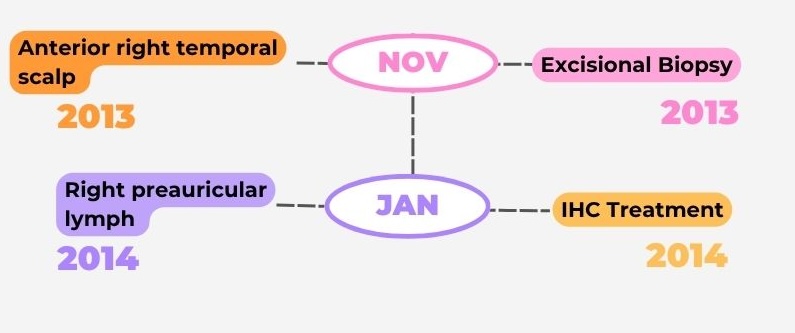


**B**. Case 2’s recurrences and treatments (2013–2014)

**Supplementary Figure 1.** Both cases’ melanoma recurrences and treatments timelines


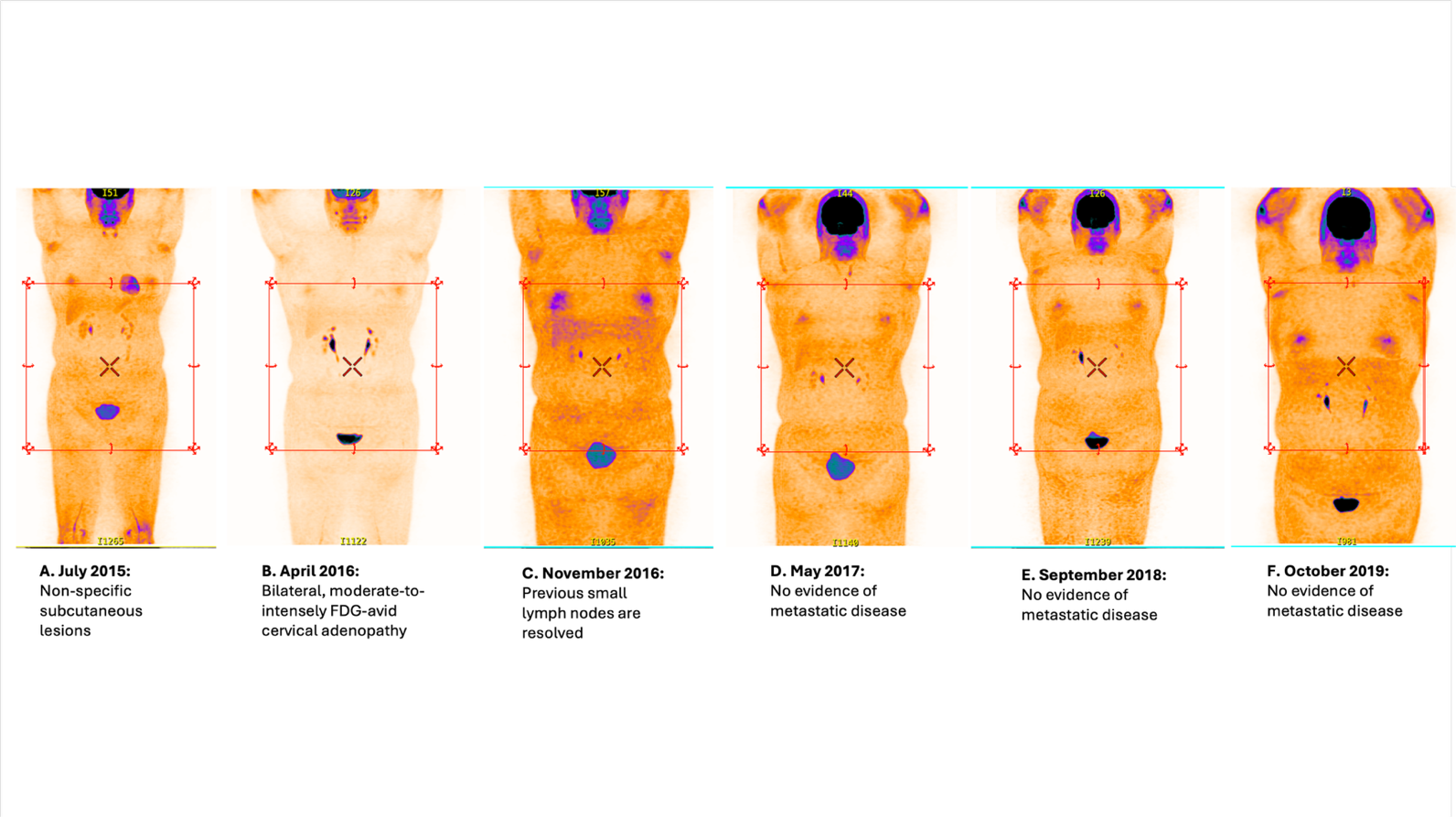


**Supplementary Figure 2.** Case 2’s serial PET scans (2015–2022) showing resolution of disease and sustained remission.
